# Associations between glycated hemoglobin and the risks of incident cardiovascular diseases in patients with gout

**DOI:** 10.1186/s12933-022-01567-9

**Published:** 2022-07-15

**Authors:** Likang Li, Gregory Y. H. Lip, Shuai Li, Jonathan D. Adachi, Lehana Thabane, Guowei Li

**Affiliations:** 1 Center for Clinical Epidemiology and Methodology (CCEM), Guangdong Second Provincial General Hospital, 510317 Guangzhou, China; 2 Liverpool Centre for Cardiovascular Science, University of Liverpool, Liverpool, UK; 3 Department of Clinical Medicine, Aalborg University, Aalborg, Denmark; 4 Department of Medicine, McMaster University, Hamilton, ON Canada; 5 Department of Health Research Methods, Evidence, and Impact (HEI), McMaster University, 1280 Main St West, Hamilton, ON L8S 4L8 Canada; 6 Centre for Evaluation of Medicines, St Joseph’s Health Care, Hamilton, ON Canada; 7 Faculty of Health Sciences, University of Johannesburg, Johannesburg, South Africa

**Keywords:** Glycated hemoglobin, Gout, Cardiovascular disease, Public health

## Abstract

**Background:**

Evidence for the relationship between glycated hemoglobin (HbA1c) levels and risk of cardiovascular diseases (CVD) in patients with gout remained sparse and limited. This study aims to explore the associations between HbA1c levels and risks of incident CVD in patients with gout.

**Methods:**

We included patients with gout who had an HbA1c measurement at baseline from the UK Biobank. CVD events were identified from through medical and death records. We used multivariable Cox proportional hazards model with a restricted cubic spline to assess the potential non-linear effect of HbA1c on CVD risk.

**Results:**

We included a total of 6,685 patients (mean age 59.7; 8.1% females) with gout for analyses. During a mean follow-up of 7.3 years, there were 1,095 CVD events documented with an incidence of 2.26 events per 100 person-years (95% confidence interval [CI]: 2.13–2.40). A quasi J-shaped association between HbA1c and risk of CVD was observed, with the potentially lowest risk found at the HbA1c of approximately 5.0% (hazard ratio [HR] = 0.65, 95% CI: 0.53–0.81). When compared with the HbAlc level of 7%, a significantly decreased risk of CVD was found from 5.0 to 6.5%, while an increased risk was observed at 7.5% (HR = 1.05) and 8.0% (HR = 1.09). Subgroup analyses yielded similar results to the main findings in general.

**Conclusions:**

Based on data from a nationwide, prospective, population-based cohort, we found a quasi J-shaped relationship between HbA1c and risk of CVD in patients with gout. More high-quality evidence is needed to further clarify the relationship between HbA1c and CVD risk in patients with gout.

**Supplementary Information:**

The online version contains supplementary material available at 10.1186/s12933-022-01567-9.

## Introduction

Gout is a common hyperuricemic metabolic disorder that causes painful inflammatory arthritis and results in a high disease burden [1]. Global data between 1990 and 2017 showed that the incidence, prevalence and economic burden of gout has continuously increased [2, 3]. Patients with gout generally have a high risk of cardiovascular comorbidities, which contributes to their increased cardiovascular mortality when compared to the general population [4].

Glycated hemoglobin (HbA1c) as a hemoglobin-glucose combination formed nonenzymatically within the cell, indicates average blood glucose concentrations over the prior 3 months [5]. Emerging studies showed that higher HbA1c levels were associated with elevated cardiovascular risk [6, 7]; however, some randomized control trials have highlighted that a low HbA1c level may not consistently yield a beneficial outcome from cardiovascular disease (CVD) events [8, 9]. Published guidelines have provided a detailed recommendation of optimal HbA1c targets for patients with diabetes mellitus [10, 11], while no guidance has been available for patients with gout. Even though current guidelines including the American College of Rheumatology, British Society for Rheumatology and European Alliance of Associations for Rheumatology all underscored the importance of glycemic control in patients with gout [12–14], more high-quality evidence is largely needed to support the adequate HbA1c target recommendation in patients with gout.

In this study, our objective was to explore the associations between HbA1c and risks of incident CVD events (coronary heart disease [CHD], stroke and CVD death) in patients with gout, aiming to provide evidence on scientific HbA1c control in relation to CVD risk. Data from the nationwide prospective United Kingdom (UK) Biobank were used for analyses in this study.

## Methods

### Participants and setting

Over 500,000 participants were recruited from the general population aged 40–69 years between 2006 and 2010 in the UK Biobank. Each participant attended one of 22 assessment centers to complete a touch-screen questionnaire, provide biological samples and have physical measurements, which had been reported in detail elsewhere [15]. This analysis was restricted to the 6,685 patients with gout who had an HbA1c measurement but did not have a CVD diagnosis at baseline. Gout was defined as either with a self-report diagnosis, the ninth revision of the International Classification of Diseases (ICD-9) code 274, or the tenth revision of the ICD (ICD-10) code M10.

The patient selection process is displayed in Additional file 1: Fig. S1 for this study. To assess the potential selection bias, we used the standardized mean difference (SMD) to examine the balance of covariate distribution between the included participants and those who were excluded from analysis in the UK biobank, where a SMD > 0.10 indicated difference in covariates between the included and excluded participants. All patients were followed up from baseline until they had a CVD event or death, or 31 March 2017, whichever came first.

 All participants provided written informed consent for participation in the research. The UK Biobank was approved by the Research Ethics Committee with a reference number of 11/NW/0382. The Guangdong Second General Provincial Hospital Research Ethics Committee approved the current analysis (2022-KY-KZ-119-01).

### Ascertainment of outcomes

Our primary outcome was a composite of incident CVD events that included CHD, stroke, and CVD death. The secondary outcomes were the individual CVD events (CHD, stroke, and CVD death).

Data on the CVD events and their timing were identified via certified death records and cumulative medical records of hospital diagnoses, all of which were linked by using the ICD-9 and the ICD-10 codes. The ICD-9 and ICD-10 codes for CHD were 410–414 and I20-I25, respectively. Stroke was identified by the 430–434 and 436 for ICD-9 and the I60-I64 for ICD-10. CVD death was defined using ICD-10 codes I00-I99.

### HbA1c and other independent variables

HbA1c was measured from frozen packed red blood cells by the Bio-Rad Variant II Turbo analyzer with high-performance liquid chromatography (Bio-Rad Lab. Inc). The unit in mmol/mol was converted to percentage (%) based on the equation: (0.09148 × HbA1c in mmol/mol) + 2.152 [16].

Data on other independent variables at baseline included age, sex, ethnicity, education, body mass index (BMI), smoking and drinking, physical activity, diabetes, hypertension, high cholesterol, osteoarthritis, rheumatoid arthritis, chronic kidney disease (CKD) and serum urate level. We also collected information on intake of non-steroidal anti-inflammatory drugs (NSAIDs), antihypertensive medications, antidiabetic medications, statins, urate-lowering drugs, and vitamin and mineral supplementation. To minimize the under-recognition of data on comorbidities and medication intake at baseline, we used the information from patients’ self-reports, the interview with trained staff regarding medications and treatment that patients received, and the ICD codes. We documented the existence of a variable if the patient had a positive response to any of the aforementioned data fields. Participants were considered to be Metabolically Healthy (MH) if they had (1) systolic blood pressure (BP) less than 130 mmHg and no use of BP-lowering medication, (2) waist-to-hip ratio (WHR) less than 0.95 (women) and less than 1.03 (men), and (3) no prevalent diabetes [17].

### Statistical analysis

Baseline variables were reported as means (standard deviations [SDs]) for continuous variables and counts (percentages) for categorical variables, respectively.

Restricted cubic splines, as commonly used to model non-linear associations in regression models, were transformation of an independent continuous variable and could be used in various regression models. The range of values of the independent variable was first split up, with “knots” defining the end of one segment and the start of the next. Subsequently, separate curves were fitted to each segment so that the resulting overall fitted curve was smooth and continuous [18]. We used the restricted cubic spline based on multivariable Cox proportional hazards model to assess the potential non-linear effect of HbA1c on CVD risk, where the HbA1c level was treated by using a restricted cubic spline with four knots laid at the 5th, 35th, 65th, and 95th percentiles. The multivariable model was adjusted for age, sex, ethnicity, education, BMI, smoking and drinking, diabetes, hypertension, high cholesterol, and osteoarthritis. We then performed general contrasts of regression coefficients for HbA1c to estimate hazard ratios (HRs) for pre-defined levels of HbA1c at 4.5, 5.0, 5.5, 6.0, 6.5, 7.5 and 8.0%, taking 7.0% as the reference. Results were presented as point estimates with their corresponding 95% confidence intervals (CIs). Similar analyses were conducted for secondary outcomes of CHD, stroke, and CVD death.

To explore whether there existed potential effect modifications, we conducted four pre-defined subgroup analyses by sex (males and females), age (< 65 and ≥ 65 years), diabetes mellitus (yes or no), and MH status (yes or no). To assess the robustness of our main findings, we carried out a series of sensitivity analysis. First, we performed a multivariable Cox model adjusting for age, sex, ethnicity, education, BMI, smoking and drinking, physical activity, diabetes, hypertension, high cholesterol, osteoarthritis, rheumatoid arthritis, CKD, NSAIDs, antihypertensive and antidiabetic medications, statins, and vitamin and mineral supplementation. Another sensitivity analysis by further adjusting for serum urate level and use of urate-lowering drugs was conducted. Moreover, we used the multivariable Cox model to estimate the associations between different HbA1c groups (< 5.0%, 5.0% to < 6.5%, and ≥ 6.5%) and CVD risk, with the HbA1c group of 5.0% to < 6.5% as reference group. Furthermore, we performed a competing risk analysis by treating death as a competing event for CVD. Finally, we used the propensity score matching method to create two pairwise-matched cohorts (HbA1c groups of < 5.0% vs. 5.0% to < 6.5%; and HbA1c groups of ≥ 6.5% vs. 5.0% to < 6.5%) based on their HbA1c levels, with the HbA1c group of 5.0% to < 6.5% as reference.

All tests were two-sided and the significance level was set as 0.05. The SAS version 9.4 (SAS Institute, Inc., Cary, NC) and R version 3.5.1 (R Foundation for Statistical Computing, Vienna, Austria) were employed for analyses.

## Results

**Fig. 1:**
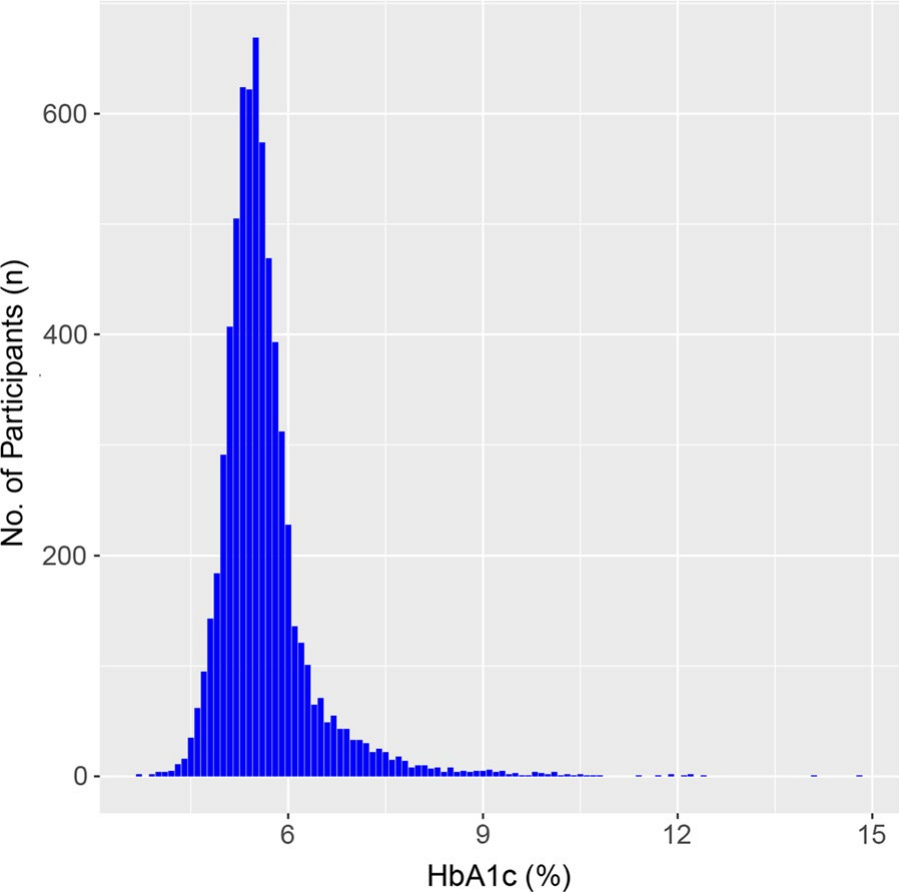
Density distribution for the HbA1c levels among all the included patients

We included a total of 6,685 patients with gout (mean age 59.7 (SD: 7.0) years; 8.1% females) for analyses. The baseline characteristics of the population are shown in Table 1. They had a mean BMI of 30.6 (SD: 4.9) kg/m^2^. Most patients were alcohol drinkers and physically active. There were 13%, 57%, 31%and 17% of the patients having diabetes, hypertension, high cholesterol and osteoarthritis, respectively. Less than 10% of the patients were MH. The mean HbA1c level was 5.6% (SD: 0.8%). As shown in Additional file 1: Table S1, some SMDs for baseline characteristics between the included and excluded participants were greater than 0.10, indicating the imbalances between the two groups and thus potential selection bias. Figure 1 displays the density distribution for the HbA1c levels among all the included patients.

**Table 1:** Description of baseline characteristics for the study participants

Characteristics	Total participants (n = 6685)
Age (years), mean (SD)	59.7 (7.0)
Sex (female), n (%)	542 (8.1)
White ethnicity, n (%)	6354 (95.4)
With college or university degree, n (%)	676 (10.2)
BMI (kg/m ^2^ ), mean (SD)	30.6 (4.9)
BMI Categories, n (%)	
Underweight (< 18.5 kg/m^2^)	1 (0.0)
Normal weight (18.5–24.9 kg/m^2^)	577 (8.7)
Overweight (25.0 to 29.9 kg/m^2^)	2846 (42.8)
Obese (≥ 30.0 kg/m^2^)	3230 (48.5)
MH status, n (%)	572 (8.6)
Smoking status, n (%)	
Never	2821 (42.4)
Previous	3214 (48.3)
Current	622 (9.3)
Alcohol intake status, n (%)	
Never	127 (1.9)
Previous	235 (3.5)
Current	6308 (94.6)
Physical activity (≥ 600 MET min per week), n (%)	4261 (77.1)
Diabetes, n (%)	845 (12.6)
Hypertension, n (%)	3823 (57.2)
High cholesterol, n (%)	2067 (30.9)
Osteoarthritis, n (%)	1131 (16.9)
Rheumatoid arthritis, n (%)	113 (1.7)
CKD, n (%)	127 (1.9)
NSAIDs, n (%)	1529 (22.9)
Antihypertensive drugs, n (%)	3,303 (49.4)
Antidiabetic medications, n (%)	615 (9.2)
Statins, n (%)	2517 (37.7)
Vitamins, n (%)	1696 (25.6)
Minerals and other dietary supplementation, n (%)	2656 (39.8)
Urate–lowering drugs	4422 (66.1)
Serum urate (umol/L), mean (SD)	379.4 (103.4)
HbA1c (%), mean (SD)	5.6 (0.8)

*SD* standard deviation, *BMI* body mass index, *MH* metabolically healthy, *MET* metabolic equivalent, *CKD* chronic kidney disease, *NSAIDs* non-steroidal anti-inflammatory drugs, *HbA1c* hemoglobin A1c

**Fig. 2:**
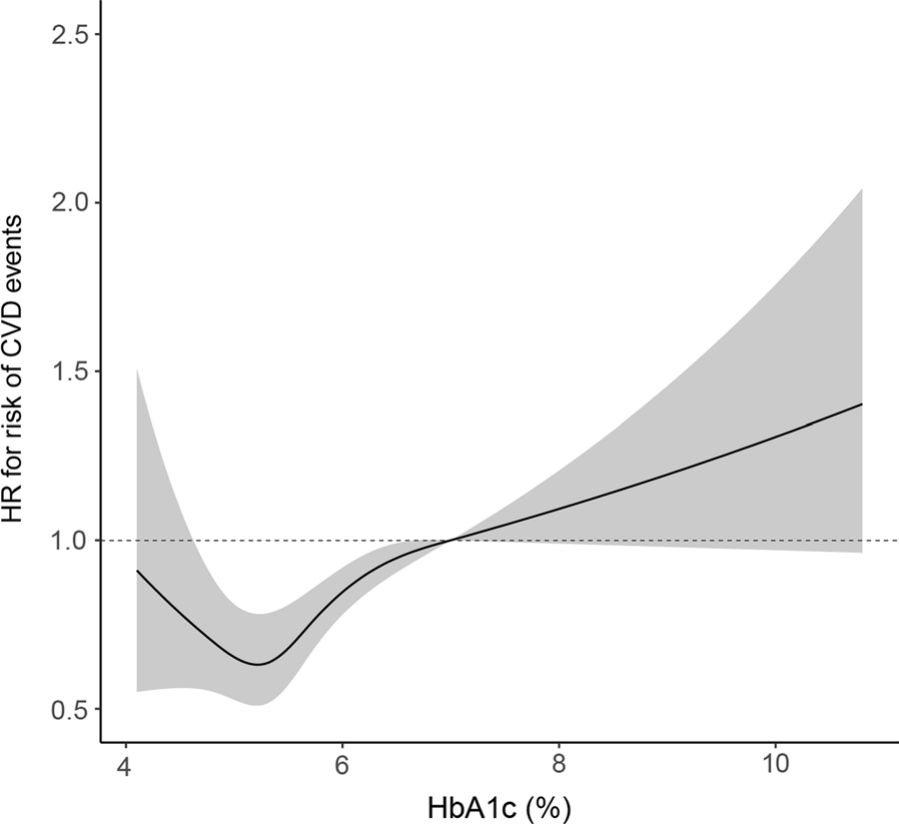
Hazard ratios for risk of CVD events in relation to different HbAlc levels (shadows indicating 95% confidence intervals for hazard ratios)

During a mean follow-up of 7.3 years, there were 1,095 CVD events documented with an incidence of 2.26 events per 100 person-years (95% CI: 2.13–2.40). Figure 2 shows the relationship between HbA1c and risk of CVD events, indicating a quasi J-shaped association with the potentially lowest CVD risk at the HbA1c level of approximately 5.0% (HR = 0.65, 95% CI: 0.53–0.81). When compared with the HbAlc level of 7%, a significantly decreased risk of CVD was found from 5.0 to 6.5% (HR = 0.95, 95% CI: 0.91–0.99), while an increased risk was observed at 7.5% (HR = 1.05, 95% CI: 1.00–1.10) and 8.0% (HR = 1.09, 95% CI: 1.00–1.21) (Table 2).

**Table 2:** Hazard Ratio (95% CI) for risks of CVD events, CHD, Stroke, CVD death at pre-defined levels of HbA1c

Outcome	No. of events/no. of patients	HbA1c (%)							
		4.5	5.0	5.5	6.0	6.5	7.0	7.5	8.0
Primary outcome									
CVD events	1095/6685	0.78 (0.56–1.09)	0.65 (0.53–0.81)	0.68 (0.57–0.81)	0.85 (0.78–0.92)	0.95 (0.91–0.99)	Ref.	1.05 (1.00–1.10)	1.09 (1.00–1.21)
Secondary outcomes									
CHD	916/6685	0.75 (0.52–1.10)	0.70 (0.55–0.89)	0.75 (0.62–0.91)	0.89 (0.81–0.98)	0.97 (0.92–1.02)	Ref.	1.03 (0.97–1.09)	1.05 (0.94–1.18)
Stroke	151/6685	1.00 (0.50–2.01)	0.53 (0.31–0.91)	0.46 (0.30–0.70)	0.64 (0.54–0.77)	0.82 (0.75–0.90)	Ref.	1.20 (1.09–1.32)	1.44 (1.19–1.76)
CVD death	144/6685	0.58 (0.22–1.53)	0.48 (0.26–0.87)	0.53 (0.33–0.85)	0.74 (0.61–0.90)	0.89 (0.81–0.99)	Ref.	1.10 (0.99–1.24)	1.22 (0.97–1.53)

*CI* confidence interval, *CVD* cardiovascular disease, *CHD* coronary heart disease, *HbA1c* hemoglobin A1c, *Ref* reference

**Table 3:** Hazard Ratio (95% CI) from subgroup analyses for risks of CVD events at pre-defined levels of HbA1c

Subgroup	No. of events/no. of patients	HbA1c (%)							
		4.5	5.0	5.5	6.0	6.5	7.0	7.5	8.0
By sex									
Male	1013/6143	0.76 (0.54–1.07)	0.64 (0.51–0.80)	0.68 (0.57–0.81)	0.85 (0.78–0.93)	0.95 (0.91–0.99)	Ref.	1.05 (0.99–1.10)	1.09 (0.98–1.21)
Female	82/542	1.38 (0.32–6.05)	1.09 (0.44–2.66)	0.90 (0.40–2.02)	0.92 (0.65–1.30)	0.96 (0.85–1.08)	Ref.	1.04 (0.91–1.20)	1.09 (0.82–1.43)
By age									
< 65 years	635/4702	0.67 (0.43–1.03)	0.64 (0.48–0.85)	0.72 (0.58–0.89)	0.87 (0.79–0.96)	0.96 (0.91–1.01)	Ref.	1.04 (0.98–1.10)	1.08 (0.96–1.21)
≥ 65 years	460/1983	0.75 (0.45–1.27)	0.57 (0.41–0.80)	0.55 (0.41–0.74)	0.76 (0.64–0.89)	0.91 (0.83–1.01)	Ref.	1.08 (0.97–1.20)	1.16 (0.94–1.44)
By diabetes mellitus									
Without diabetes	831/5840	0.86 (0.59–1.25)	0.69 (0.55–0.87)	0.76 (0.63–0.92)	0.92 (0.79–1.07)	0.96 (0.89–1.04)	Ref.	1.04 (0.96–1.12)	1.08 (0.93–1.26)
With diabetes	264/845	1.00 (0.42–2.36)	0.94 (0.53–1.65)	0.88 (0.64–1.22)	0.85 (0.65–1.11)	0.90 (0.76–1.06)	Ref.	1.10 (1.01–1.20)	1.20 (1.05–1.38)
By MH status									
MH	46/572	0.60 (0.10–3.53)	0.39 (0.10–1.51)	0.44 (0.11–1.77)	0.59 (0.20–1.74)	0.77 (0.45–1.32)	Ref.	1.30 (0.76–2.23)	1.70 (0.58–5.00)
MU	1039/6088	0.61 (0.44–0.85)	0.50 (0.42–0.60)	0.53 (0.46–0.62)	0.76 (0.71–0.82)	0.92 (0.88–0.96)	Ref.	1.06 (1.01–1.11)	1.13 (1.03–1.24)

*CI* confidence interval, *CVD* cardiovascular disease, *HbA1c* hemoglobin A1c, *MH* metabolically healthy, *MU* metabolically unhealthy, *Ref* reference

We observed 916 CHD events during follow-up (incidence: 1.88 events per 100 person-years, 95% CI: 1.76–2.00). The levels of HbA1c between 5.0% and 6.0% was significantly associated with a reduced risk of CHD when compared with 7%, while an increased CHD risk was found at 7.5% and 8.0%. An HbA1c level of approaching 5.0% was found to have the potentially lowest CHD risk (HR = 0.70, 95% CI: 0.55–0.89; Table 2, Additional file 1: Fig. S2a). There were 151 stroke events (incidence: 0.29 events per 100 person-years, 95% CI: 0.25–0.34) and 144 CVD deaths (incident rate 0.27 events per 100 person-years, 95% CI: 0.23–0.32) found during follow-up. Similarly, an approximately J-shaped relationship between HbAlc levels and risks of stroke and CVD deaths was also observed (Additional file 1: Fig. S2b, c). The lowest risk of stroke was found at the HbAlc level of 5.5% (HR = 0.46, 95% CI: 0.30–0.70), while the level of approximately 5.0% was associated with the largest reduction in risk of CVD death (HR = 0.48, 95% CI: 0.26–0.87) (Table 2).

Subgroup analyses yielded in general similar results to the main findings **(**Table 3, Additional file 1: Figs. S3–6). For males, patients aged < 65 years, without diabetes and with a MU status, the potentially lowest CVD risk was observed at the HbA1c level of about 5.0% (HRs ranging from 0.50 to 0.69). The lowest CVD risk for age ≥ 65 years (HR = 0.55) was observed at the HbAlc level of approaching 5.5%. A non-significant inflection point was found at HbA1c of 6.0% (HR = 0.85, 95% CI: 0.65–1.11). No obvious J-shaped associations were detected in other subgroups.

### Sensitivity analyses

Similar trends from sensitivity analyses were found regarding HbA1c and risk of incident CVD, CHD, stroke and CVD death in patients with gout when different covariates were adjusted for **(**Additional file 1: Figs. S7 and S8**)**. Results for the three HbA1c groups (< 5.0%, 5.0% to < 6.5%, and ≥ 6.5%) were shown in Additional file 1: Table S2, where the CVD risks were significantly elevated in the HbA1c groups of < 5.0% and ≥ 6.5% when compared with the HbA1c group of 5.0% to < 6.5%.

## Discussion

In this study, we found a quasi J-shaped relationship between HbA1c level and risk of CVD events in patients with gout. When compared with 7%, HbA1c across the range of 5.0–6.5% was associated with a 5–35% lower risk of CVD events in patients with gout, where the potentially largest reduction in CVD risk laid at the HbA1c level of approximately 5.0%.

Increased HbA1c levels were generally associated with elevated CVD risk [6, 7, 19], while lower levels of HbA1c may not consistently relate with a beneficial CVD outcome [20, 21]. In our study, we demonstrated an approximately J-shaped association between HbA1c levels and risk of CVD amongst patients with gout with the inflection point of approaching 5.0%. This observation was in line with a previous report based on 73 prospective studies involving 294,998 participants demonstrating a J-shaped association between HbA1c levels and CVD risk for individuals *without a history of diabetes mellitus or CVD at baseline*, with patients of HbA1c 5.0-5.5% having the lowest risk [20]. In another study, a J-shaped association between HbA1c and CVD risk was also observed among patients *with type 2 diabetes mellitus*; however the HbA1c level of 6.5–6.9% was related with the lowest risk [22]. Therefore, the HbA1c levels associated with the lowest CVD risk in patients with type 2 diabetes mellitus were higher than in patients with gout from our study. Potential interpretation may be due to the fact that tighter control of HbA1c could help mitigate the inflammatory response and thus be related with favorable CVD outcomes in patients with gout [4, 23]. Furthermore, hypoglycemia was a common complication in diabetes and intensive HbA1c targets could increase the risk of hypoglycemia, especially for those treated with insulin [24, 25]. One study showed no evidence of cardiovascular benefit from tighter glycemic control (an HbA1c level of 6.5% or lower) compared with standard care among patients with diabetes [26]. Another trial also exhibited that intensive treatment (HbA1c level of < 6.0%) in patients with diabetes increased the risk of death when compared with an HbA1c level of 7.0–7.9% [8].

While the fact that risks of tight glycemic control may outweigh its benefits in patients with diabetes required further exploration, our results demonstrated that a tight glycemic control target (HbA1c level of 5.0-6.5%) in patients with gout may be significantly related with the potential benefits for CVD prevention. However, additional studies taking into account the differences in patient characteristics and physiopathology are needed to further clarify whether the glycemic control targets should be different between patients with gout and with diabetes. Nevertheless, our findings may indicate that either a low or high level of HbA1c was associated with elevated risk of CVD and therefore may provide some evidence about the HbA1c ranges in relation to CVD prevention in patients with gout.

Approximately J-shaped associations between HbA1c levels and risks of CHD, stroke and CVD death were also observed amongst patients with gout. The lowest risks of CHD and CVD death were observed at the HbA1c of approaching 5.0%, while the lowest risk of stroke was observed at the HbA1c of nearly 5.5%. Among the community-based population of patients without diabetes, one study showed that higher HbA1c levels were significantly related with increased risks of CHD and stroke, while the associations were linear [27]. The study also observed a J-shaped pattern of association between HbA1c and risk of death from any cause, with the lowest death risk at the HbA1c level of 5.0–5.5% [27]. Our different association patterns and inflection points from this published study may in part be due to the heterogeneous populations and the different outcome definitions. Nonetheless, our results for these secondary outcomes required more adequately-powered and well-designed studies for further clarification.

The potentially lowest risk of CVD was found at HbA1c of 6.0% in patients with gout and diabetes, which was higher than the inflection point (5.0%) in the overall patients with gout. This may indicate that a less stringent glycemic control target than in the overall gout population, but a more intensive target than the recommendation for the general diabetic patients, was needed for those patients with gout and diabetes. Few studies have described whether MH could modify the relationship between HbA1c and CVD risk in patients with gout. We found a quasi J-shaped association between HbA1c and CVD risk amongst MU patients, rather than in MH patients. This may be because of the relatively small sample size of MH patients resulting in insufficient statistical power. However, more investigation was required for further exploration given the exploratory and hypothesis-generating nature of these subgroup analyses.

Our findings emphasized the importance of maintaining an adequate level of HbA1c for prevention of CVD in patients with gout. In patients with gout, systemic inflammation was substantially associated with an increased risk of CVD [4]. While inflammation has been associated with higher levels of HbA1c [23], good control of HbA1c may help mitigate the inflammatory response and thus be related with decreased risk of CVD in patients with gout. Moreover, gout has been shown to be an independent risk factor for CVD [4, 28, 29]. For example, data from > 51,000 men in the Health Professionals Follow-Up Study showed that after adjusting for traditional risk factors including diabetes, hypertension and hyperlipidemia, men with gout had a 28% higher all-cause mortality and 38% higher cardiovascular mortality when compared with those without gout [30]. Therefore, it may be possible for patients with gout to control their CVD risk by defining a precise HbA1c control target and providing specific recommendations. However, no specific recommendations for their glycemic targets were clearly given for patients with gout from current guidelines [12–14, 31]. Thus, our results may provide some insights into the adequate HbA1c levels in relation to CVD prevention in patients with gout.

## Strengths and limitations

This study has several strengths. First, the UK Biobank is a nationwide, prospective, population-based cohort with a large sample size and long-term follow-up. We modeled HbA1c as a continuous exposure variable via the non-linear analysis and presented data mainly in a graphical format, which could help with easy and straightforward understanding. Rigorous methodology and detailed analyses also supported the validity of our results. Several limitations exist in this study. As an observational study without a randomized design, we could not fully preclude confounding effects especially of those unmeasured variables, which may compromise the credibility and strength of our results [32]. For instance, the observed associations between HbA1c and CVD risks may be driven by some unmeasured factors related with frailty and lifestyle, which would impair our findings to an unknown extent. HbA1c was measured only at baseline and may change over time, which could influence the patients’ subsequent CVD risk. Unfortunately, we could not assess the change in HbA1c in relation to CVD risk due to the data unavailability. The usage of linked data on medical and death reports to ascertain the CVD events may underestimate the true incidence due to the existence of subclinical episodes of CVD events. It had been argued that the UK Biobank consisted of relatively healthy participants, which may therefore limit the generalizability of our findings to populations with comorbidities [33]. Some imbalances for baseline characteristics between the included and excluded participants were found; this thus indicated a potential selection bias for our study participants and would impair the validity and generalizability of the study findings to an unknown extent. Therefore our results should be interpreted with caution, requiring more research to further verify the association between HbA1c levels and CVD risk in patients with gout.

## Conclusions

Based on data from a nationwide, prospective, population-based cohort, we found a quasi J-shaped relationship between HbA1c and risk of CVD events in patients with gout, where the potentially lowest point was found at HbA1c level of approximate 5.0%. These results may provide some evidence about the adequate HbA1c levels in relation to prevention of CVD. More high-quality evidence is needed to further clarify the relationship between HbA1c and CVD risk in patients with gout.

## Supplementary Information

**Additional file 1: Fig. S1.** Flow diagram showing the selection of participants in this study. **Table S1.** Comparisons of baseline characteristics between included and excluded participants in UK biobank. **Table S2.** Results from sensitivity analyses for the relationship between different HbA1c groups and risk of CVD. **Fig. S2.** Hazard ratios for risk of CHD, stroke, and CVD death in relation to different HbAlc levels (shadows indicating 95% confidence intervals for hazard ratios). **Fig. S3.** Hazard ratios for risk of CVD events in relation to different HbAlc levels stratified by *sex* (shadows indicating 95% confidence intervals for hazard ratios). **Fig. S4. **Hazard ratios for risk of CVD events in relation to different HbAlc levels stratified by *age group* (shadows indicating 95% confidence intervals for hazard ratios). **Fig. S5.** Hazard ratios for risk of CVD events in relation to different HbAlc levels stratified by *diabetes* (shadows indicating 95% confidence intervals for hazard ratios). **Fig. S6.** Hazard ratios for risk of CVD events in relation to different HbAlc levels stratified by *MH status* (shadows indicating 95% confidence intervals for hazard ratios). **Fig. S7.** Sensitivity analysis results of hazard ratios for risk of CVD events, CHD, stroke, and CVD death in relation to different HbAlc levels (shadows indicating 95% confidence intervals for hazard ratios). **Fig. S8.** Sensitivity analysis results of hazard ratios for risk of CVD events, CHD, stroke, and CVD death in relation to different HbAlc levels (models further adjusted for urate-lowering drugs and serum urate, shadows indicating 95% confidence intervals for hazard ratios)

## Data Availability

The data can be available on application to the UK Biobank (www.ukbiobank.ac.uk/). Data described for the analyses and in the manuscript will be made available upon request.

## Abbreviations

HbA1c: Glycated hemoglobin
CVD: Cardiovascular diseases
CHD: Coronary heart disease
UK: United Kingdom
ICD: International Classification of Diseases
BMI: Body mass index
CKD: Chronic kidney disease
NSAIDs: Non-steroidal anti-inflammatory drugs
MH: Metabolically Health
BP: Blood pressure
WHR: Waist-to-hip ratio
SDs: Standard deviations
SMD: Standardized mean difference
CIs: Confidence intervals
HRs: Hazard ratios
