# Supplementary material for: Associations between glycated hemoglobin and the risks of incident cardiovascular diseases in patients with gout
Source: Cardiovasc Diabetol. 2022 Jul 15;21:133. doi: 10.1186/s12933-022-01567-9 (PMC9284835; doi:10.1186/s12933-022-01567-9)

**Supplemental data**

**List of contents:**

[Supplemental Figure 1. Flow diagram showing the selection of participants in this study. 2](#_Toc107846071)

[Supplemental Table 1. Comparisons of baseline characteristics between included and excluded participants in UK biobank 3](#_Toc107846072)

[Supplemental Table 2. Results from sensitivity analyses for the relationship between different HbA1c groups and risk of CVD. 4](#_Toc107846073)

[Supplemental Figure 2. Hazard ratios for risk of CHD, stroke, and CVD death in relation to different HbAlc levels (shadows indicating 95% confidence intervals for hazard ratios) 5](#_Toc107846074)

[Supplemental Figure 3. Hazard ratios for risk of CVD events in relation to different HbAlc levels stratified by sex (shadows indicating 95% confidence intervals for hazard ratios) 6](#_Toc107846075)

[Supplemental Figure 4. Hazard ratios for risk of CVD events in relation to different HbAlc levels stratified by age group (shadows indicating 95% confidence intervals for hazard ratios) 7](#_Toc107846076)

[Supplemental Figure 5. Hazard ratios for risk of CVD events in relation to different HbAlc levels stratified by diabetes (shadows indicating 95% confidence intervals for hazard ratios) 8](#_Toc107846077)

[Supplemental Figure 6. Hazard ratios for risk of CVD events in relation to different HbAlc levels stratified by MH status (shadows indicating 95% confidence intervals for hazard ratios) 9](#_Toc107846078)

[Supplemental Figure 7. Sensitivity analysis results of hazard ratios for risk of CVD events, CHD, stroke, and CVD death in relation to different HbAlc levels (shadows indicating 95% confidence intervals for hazard ratios) 10](#_Toc107846079)

[Supplemental Figure 8. Sensitivity analysis results of hazard ratios for risk of CVD events, CHD, stroke, and CVD death in relation to different HbAlc levels (models further adjusted for urate-lowering drugs and serum urate, shadows indicating 95% confidence intervals for hazard ratios) 11](#_Toc107846080)

# Supplemental Figure 1. Flow diagram showing the selection of participants in this study.

502,493

Total UK Biobank participants

Excluding 495,060 participants not with a diagnosis of gout by a physician

7,433 participants

Excluding 299 participants with a CVD diagnosis at baseline

Excluding 449 participants with missing data on HbA1c

6,685 participants were included for analyses

# Supplemental Table 1. Comparisons of baseline characteristics between included and excluded participants in UK biobank

| **Characteristics** | **Participants included (n = 6,685)** | **Participants excluded (n = 495,808)** | **SMD** |
| --- | --- | --- | --- |
| **Age (years)**, mean (SD) | 59.7 (7.0) | 56.5 (8.1) | 0.42 |
| **Sex (female)**, n (%) | 542 (8.1) | 272,835 (55.0) | 1.17 |
| **White ethnicity**, n (%) | 6,354 (95.4) | 466,329 (94.6) | 0.04 |
| **With college or university degree**, n (%) | 676 (10.2) | 56,215 (11.6) | 0.04 |
| **BMI (kg/m^2^)**, mean (SD) | 30.6 (4.9) | 27.4 (4.8) | 0.66 |
| **BMI Categories**, n (%) | | | |
| Underweight (< 18.5 kg/m2) | 1 (0.0) | 2,496 (0.5) | 0.71 |
| Normal weight (18.5 – 24.9 kg/m2) | 577 (8.7) | 159,403 (32.4) |  |
| Overweight (25.0 to 29.9 kg/m^2^) | 2,846 (42.8) | 210,494 (42.7) |  |
| Obese (≥ 30.0 kg/m^2^) | 3,230 (48.5) | 120,341 (24.4) |  |
| **MH status**, n (%) | 572 (8.6) | 150,729 (30.6) | 0.58 |
| **Smoking status**, n (%) | | | |
| Never | 2,821 (42.4) | 270,695 (54.9) | 0.29 |
| Previous | 3,214 (48.3) | 169,837 (34.5) |  |
| Current | 622 (9.3) | 52,355 (10.6) |  |
| **Alcohol intake status**, n (%) | | | |
| Never | 127 (1.9) | 22,258 (4.5) | 0.15 |
| Previous | 235 (3.5) | 17,867 (3.6) |  |
| Current | 6,308 (94.6) | 454,044 (91.9) |  |
| **Physical activity (≥ 600 MET min per week)**, n (%) | 4,261 (77.1) | 322,469 (81.3) | 0.10 |
| **Diabetes**, n (%) | 845 (12.6) | 23,228 (4.7) | 0.29 |
| **Hypertension**, n (%) | 3,823 (57.2) | 132,719 (26.8) | 0.65 |
| **High cholesterol**, n (%) | 2,067 (30.9) | 67,221 (13.6) | 0.43 |
| **Osteoarthritis**, n (%) | 1,131 (16.9) | 52,254 (10.5) | 0.19 |
| **Rheumatoid arthritis**, n (%) | 113 (1.7) | 6,366 (1.3) | 0.03 |
| **CKD**, n (%) | 127 (1.9) | 829 (0.2) | 0.17 |
| **NSAIDs**, n (%) | 1,529 (22.9) | 74,023 (14.9) | 0.20 |
| **Antihypertensive drugs**, n (%) | 3,303 (49.4) | 100,692 (20.3) | 0.64 |
| **Antidiabetic medications**, n (%) | 615 (9.2) | 18,550 (3.7) | 0.22 |
| **Statins**, n (%) | 2,517 (37.7) | 79,431 (16.0) | 0.50 |
| **Vitamins**, n (%) | 1,696 (25.6) | 156,223 (32.0) | 0.14 |
| **Minerals and other dietary supplementation**, n (%) | 2,656 (39.8) | 211,129 (43.1) | 0.07 |
| **Urate-lowering drugs** | 4,422 (66.1) | 1,252 (0.3) | 1.96 |
| **Serum urate (umol/L)**, mean (SD) | 379.4 (103.4) | 308.2 (79.6) | 0.77 |
| **HbA1c (%)**, mean (SD) | 5.6 (0.8) | 5.5 (0.6) | 0.26 |

SMD: standardized mean difference; SD: standard deviation; BMI: body mass index; MH: metabolically healthy; MET: metabolic equivalent; CKD: chronic kidney disease; NSAIDs: non-steroidal anti-inflammatory drugs; HbA1c: hemoglobin A1c

# Supplemental Table 2. Results from sensitivity analyses for the relationship between different HbA1c groups and risk of CVD.

| **Analysis^1^** | **CVD events** | | |
| --- | --- | --- | --- |
|  | **No. of patients/**  **no. of CVD events** | **HR (95% CI)** | **P-value** |
| **Cox proportional hazards model^2^** | | | |
| Group 1 | 563/74 | 1.31 (1.01 – 1.71) | 0.045 |
| Group 2 | 5517/828 | Ref | - |
| Group 3 | 605/193 | 1.44 (1.13 – 1.85) | 0.004 |
| **Competing risk analysis^3^** | | | |
| Group 1 | 563/74 | 1.31 (1.00 – 1.71) | 0.048 |
| Group 2 | 5517/828 | Ref | - |
| Group 3 | 605/193 | 1.43 (1.12 – 1.84) | 0.005 |
| **Propensity score matching^4^** | | | |
| **Group 1 vs Group 2** | | | |
| Group 2 | 550/52 | Ref | - |
| Group 1 | 550/74 | 1.48 (1.04 – 2.11) | 0.031 |
| **Group 3 vs Group 2** | | | |
| Group 2 | 586/128 | Ref | - |
| Group 3 | 586/183 | 1.55 (1.24 – 1.95) | < 0.001 |

CVD: cardiovascular disease; HR: hazard ratio; CI: confidence interval; Ref: reference

^1^ HbA1c levels of < 5.0% for group 1, HbA1c levels of 5.0% to < 6.5% for group 2, HbA1c levels of ≥ 6.5% for group 3

^2^ Model adjusted for age, sex, ethnicity, education, BMI, smoking and drinking, physical activity, diabetes, hypertension, high cholesterol, osteoarthritis, rheumatoid arthritis, CKD, NSAIDs, antihypertensive and antidiabetic medications, statins, vitamin and mineral supplementation, urate-lowering drugs, and serum urate

^3^ Analysis by treating death as a competing event

^4^ Propensity score matching method was used to create two pairwise-matched cohorts based on their HbA1c levels, then performed further survival analysis.

# Supplemental Figure 2. Hazard ratios for risk of CHD, stroke, and CVD death in relation to different HbAlc levels (shadows indicating 95% confidence intervals for hazard ratios)


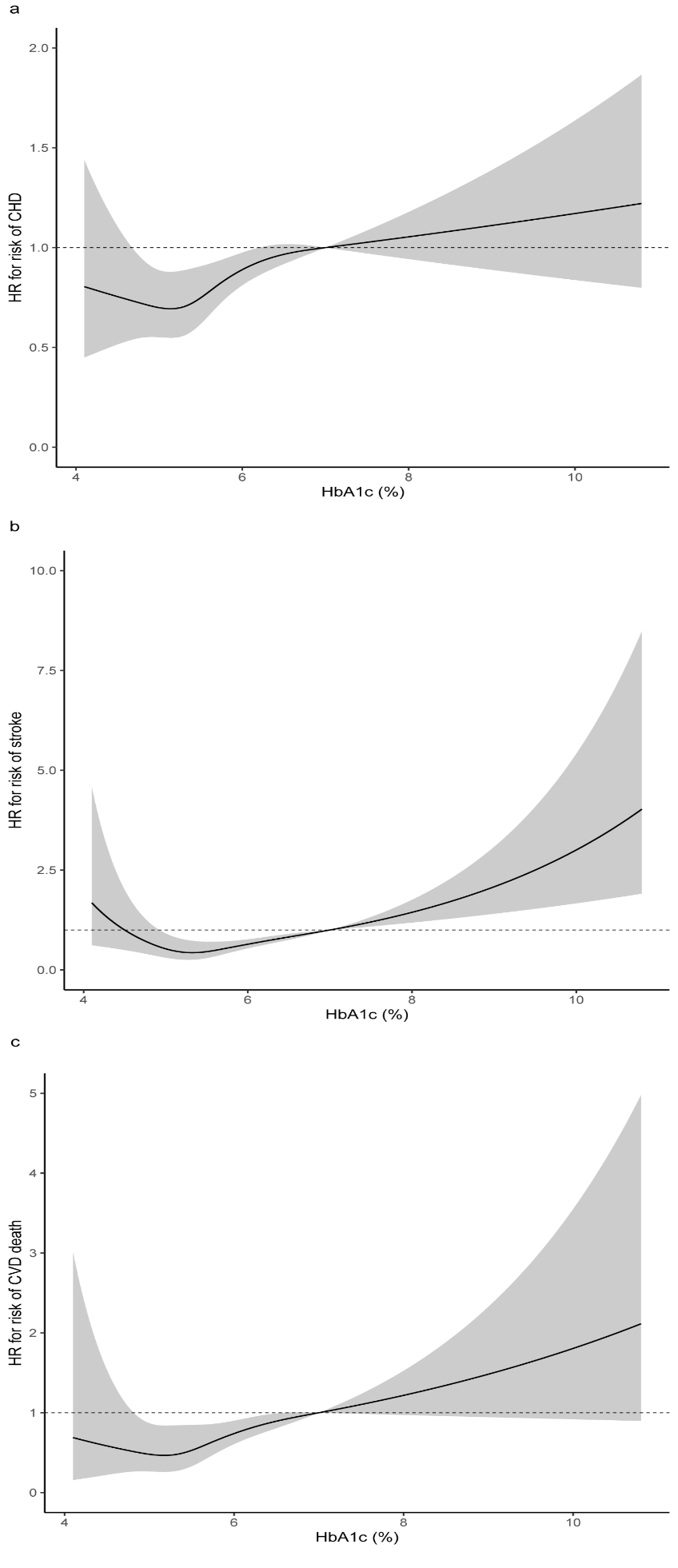


# Supplemental Figure 3. Hazard ratios for risk of CVD events in relation to different HbAlc levels stratified by sex (shadows indicating 95% confidence intervals for hazard ratios)


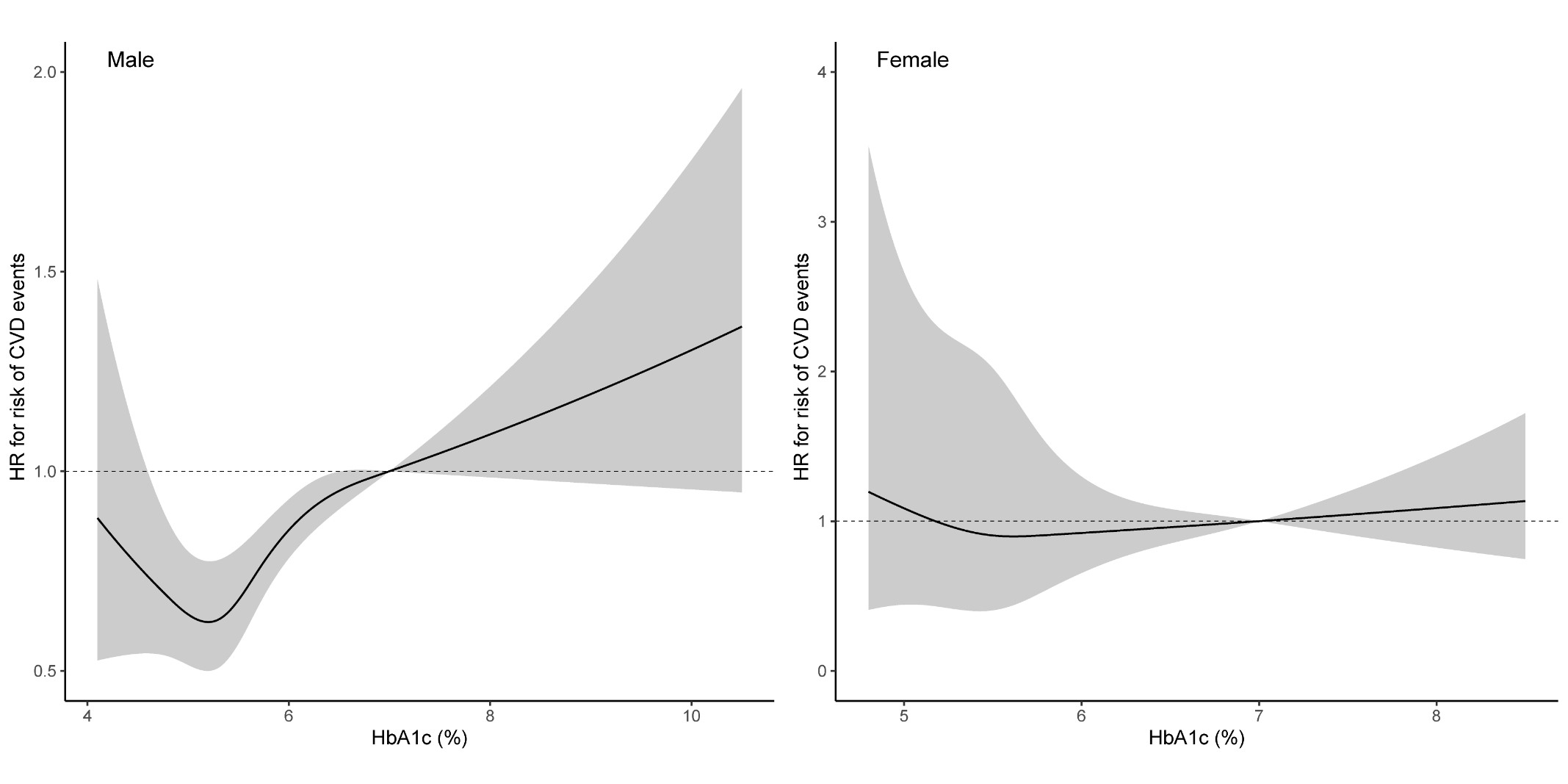


# Supplemental Figure 4. Hazard ratios for risk of CVD events in relation to different HbAlc levels stratified by age group (shadows indicating 95% confidence intervals for hazard ratios)


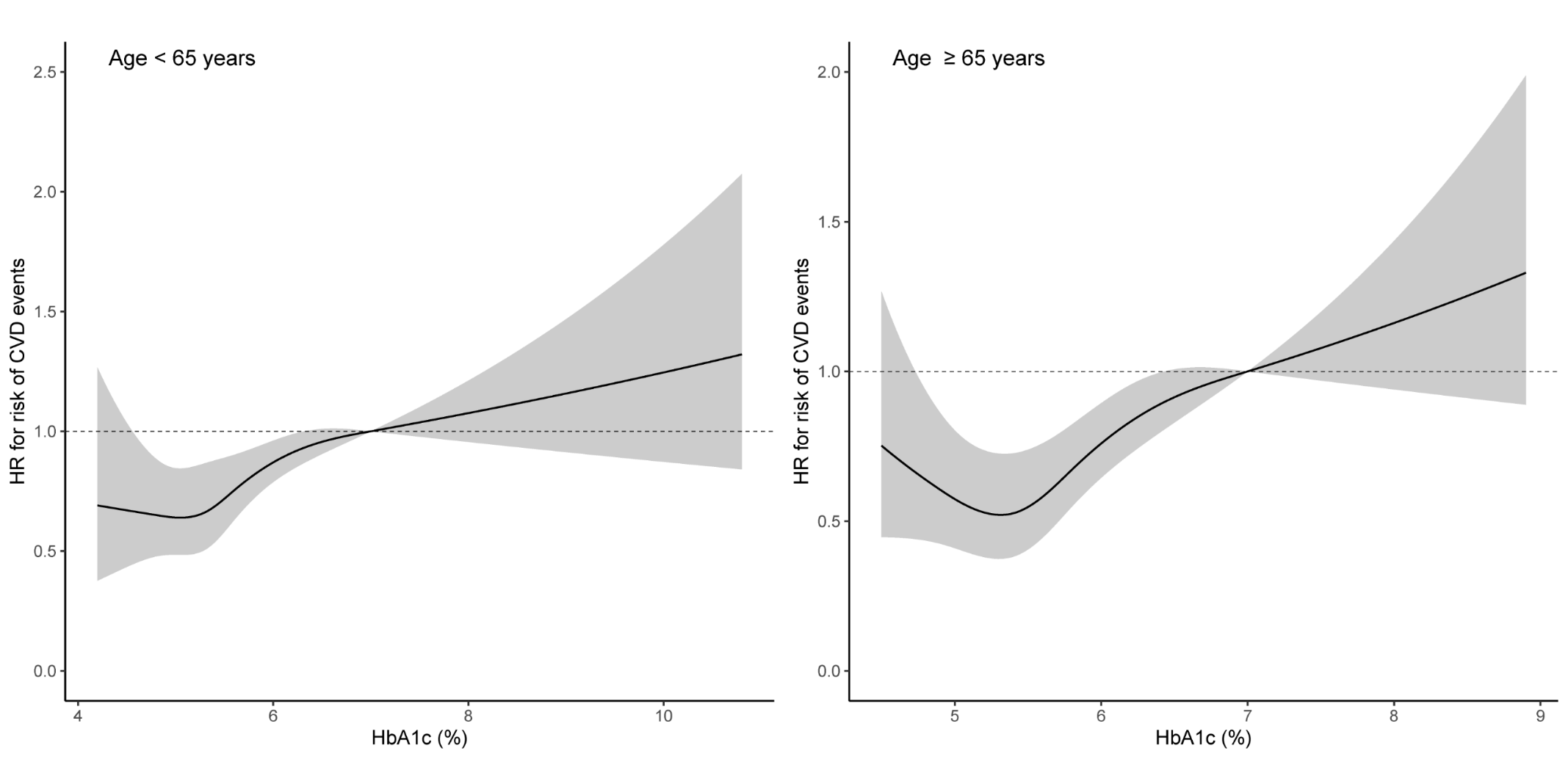


# Supplemental Figure 5. Hazard ratios for risk of CVD events in relation to different HbAlc levels stratified by diabetes (shadows indicating 95% confidence intervals for hazard ratios)


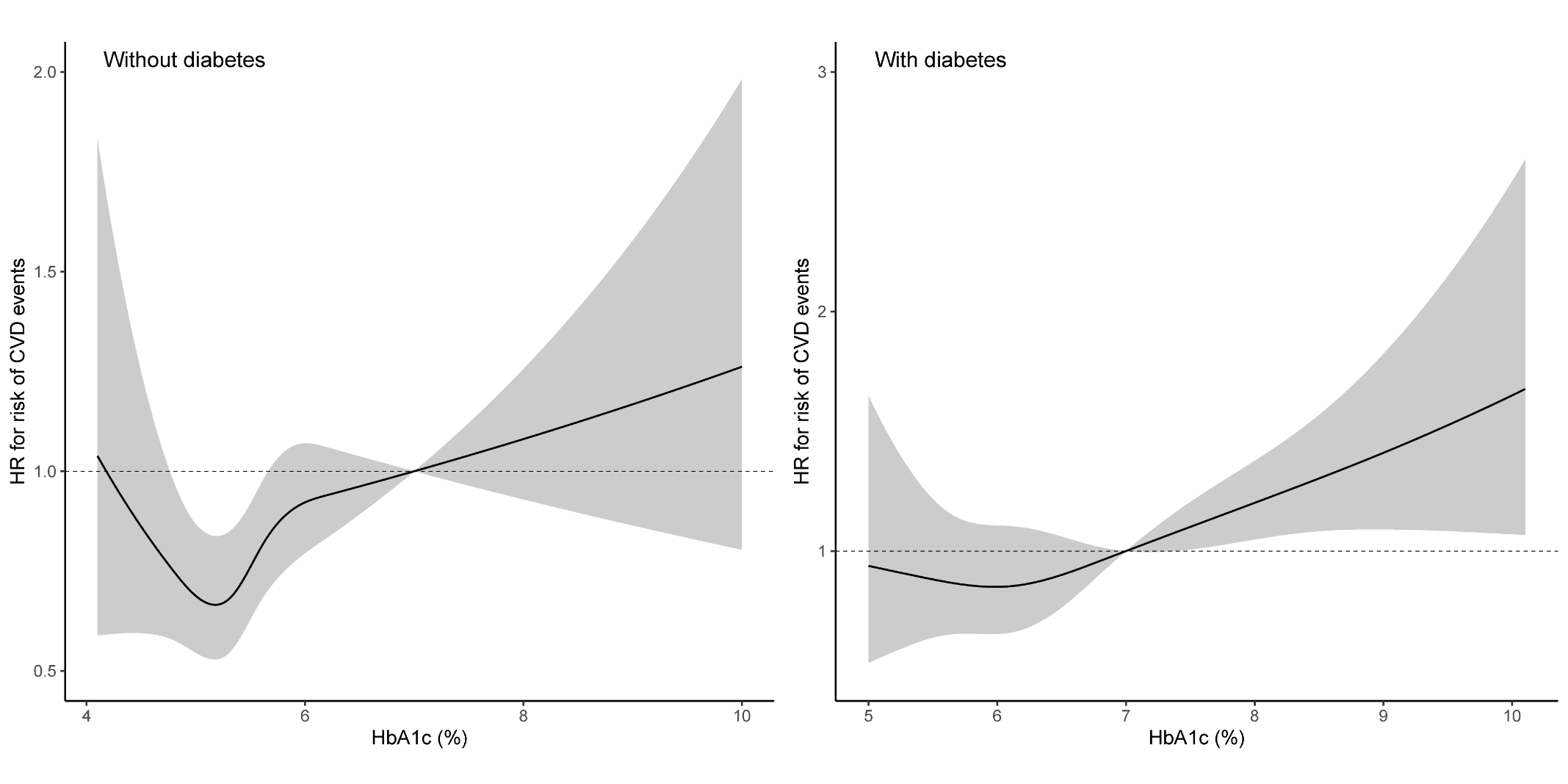


# Supplemental Figure 6. Hazard ratios for risk of CVD events in relation to different HbAlc levels stratified by MH status (shadows indicating 95% confidence intervals for hazard ratios)


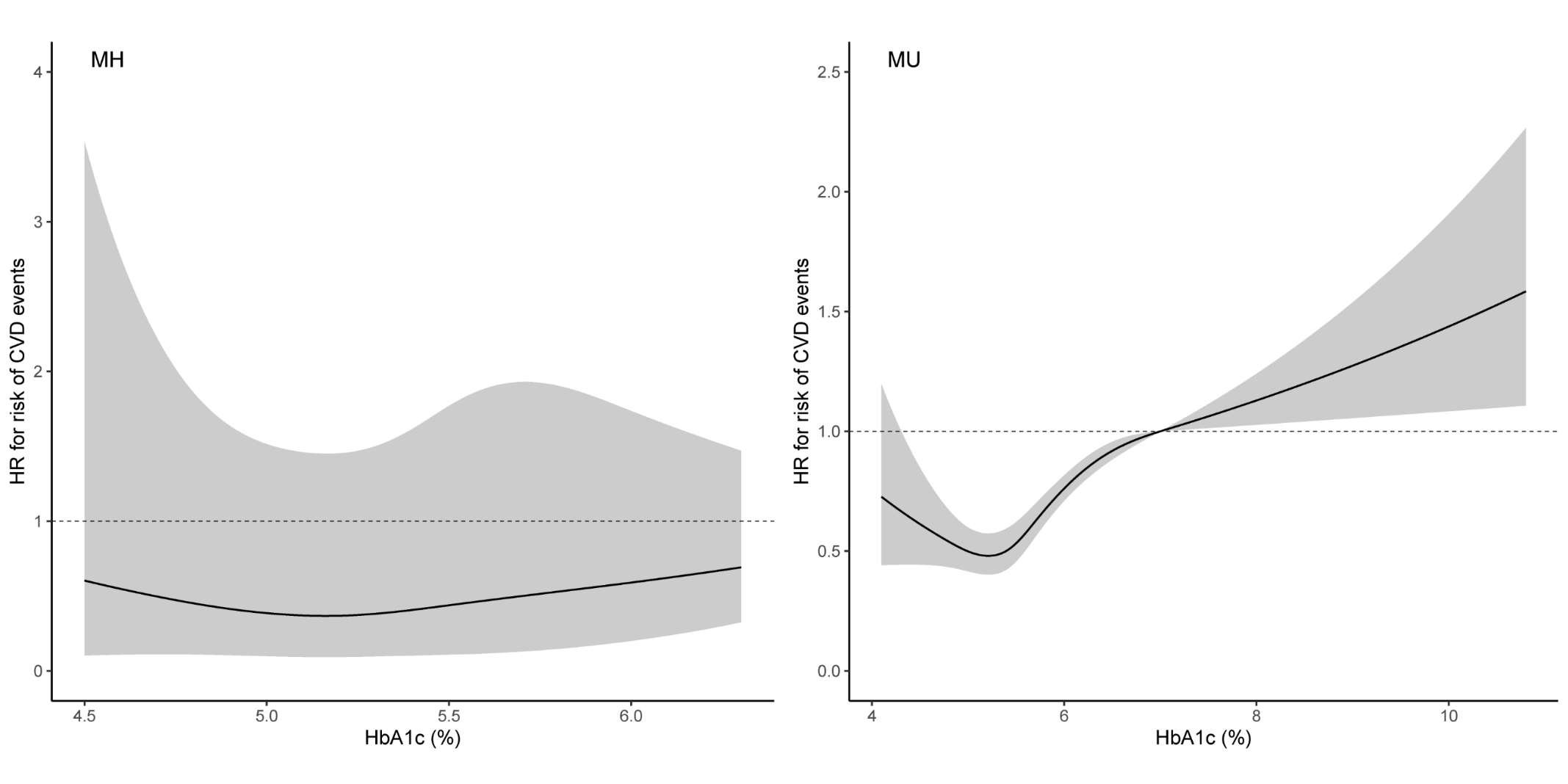


* MH = Metabolically Healthy; MU = Metabolically Unhealthy

# Supplemental Figure 7. Sensitivity analysis results of hazard ratios for risk of CVD events, CHD, stroke, and CVD death in relation to different HbAlc levels (shadows indicating 95% confidence intervals for hazard ratios)


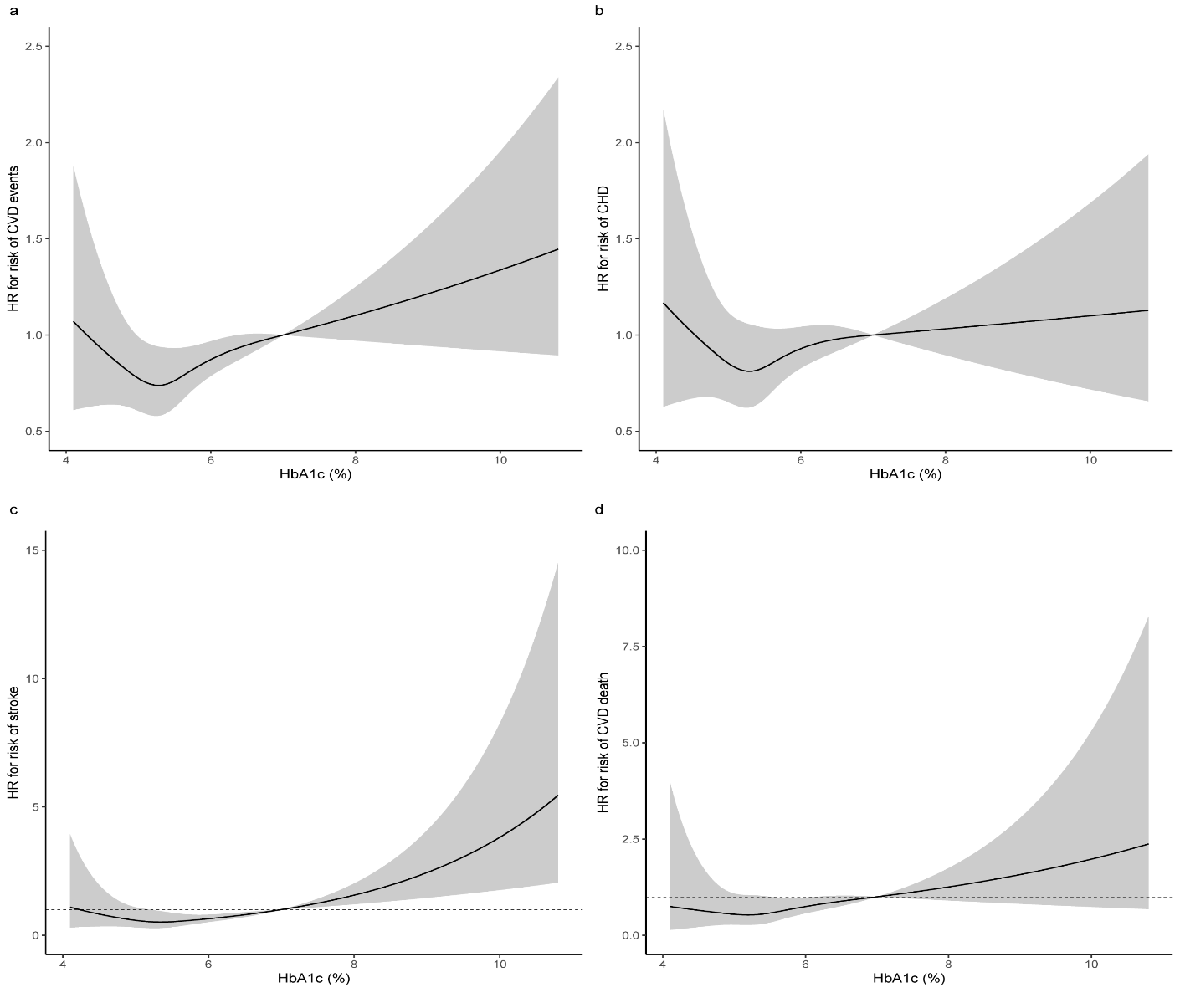


# Supplemental Figure 8. Sensitivity analysis results of hazard ratios for risk of CVD events, CHD, stroke, and CVD death in relation to different HbAlc levels (models further adjusted for urate-lowering drugs and serum urate, shadows indicating 95% confidence intervals for hazard ratios)


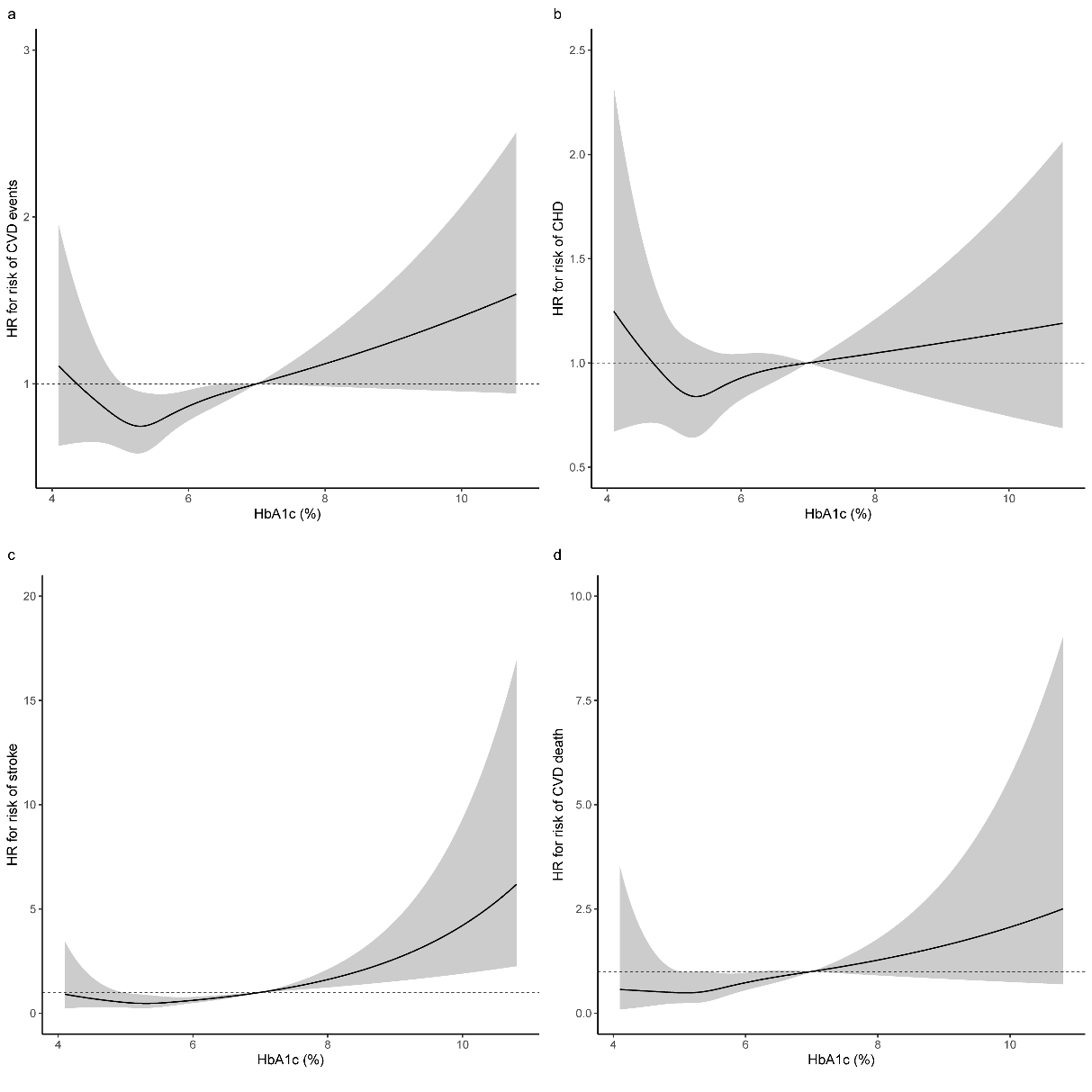

Supplement: Supplementary file 1 — Additional file 1: Fig. S1. Flow diagram showing the selection of participants in this study. Table S1. Comparisons of baseline characteristics between included and excluded participants in UK biobank. Table S2. Results from sensitivity analyses for the relationship between different HbA1c groups and risk of CVD. Fig. S2. Hazard ratios for risk of CHD, stroke, and CVD death in relation to different HbAlc levels (shadows indicating 95% confidence intervals for hazard ratios). Fig. S3. Hazard ratios for risk of CVD events in relation to different HbAlc levels stratified by sex (shadows indicating 95% confidence intervals for hazard ratios). Fig. S4. Hazard ratios for risk of CVD events in relation to different HbAlc levels stratified by age group (shadows indicating 95% confidence intervals for hazard ratios). Fig. S5. Hazard ratios for risk of CVD events in relation to different HbAlc levels stratified by diabetes (shadows indicating 95% confidence intervals for hazard ratios). Fig. S6. Hazard ratios for risk of CVD events in relation to different HbAlc levels stratified by MH status (shadows indicating 95% confidence intervals for hazard ratios). Fig. S7. Sensitivity analysis results of hazard ratios for risk of CVD events, CHD, stroke, and CVD death in relation to different HbAlc levels (shadows indicating 95% confidence intervals for hazard ratios). Fig. S8. Sensitivity analysis results of hazard ratios for risk of CVD events, CHD, stroke, and CVD death in relation to different HbAlc levels (models further adjusted for urate-lowering drugs and serum urate, shadows indicating 95% confidence intervals for hazard ratios) [file 12933_2022_1567_MOESM1_ESM.docx]
